# Autologous Periosteum-Derived Micrografts and PLGA/HA Enhance the Bone Formation in Sinus Lift Augmentation

**DOI:** 10.3389/fcell.2017.00087

**Published:** 2017-09-27

**Authors:** Ruggero Rodriguez y Baena, Riccardo D'Aquino, Antonio Graziano, Letizia Trovato, Antonio C. Aloise, Gabriele Ceccarelli, Gabriella Cusella, André A. Pelegrine, Saturnino M. Lupi

**Affiliations:** ^1^Department of Clinical Surgical, Diagnostic and Pediatric Sciences, University of Pavia, Pavia, Italy; ^2^Private Practice, Turin, Italy; ^3^Human Brain Wave S.r.L., Turin, Italy; ^4^Sbarro Health Research Organization (SHRO), Temple University of Philadelphia, Philadelphia, PA, United States; ^5^Department of Implantology, São Leopoldo Mandic Institute and Research Center, Campinas, Brazil; ^6^Department of Public Health, Experimental Medicine and Forensics, University of Pavia, Pavia, Italy; ^7^Centre for Health Technologies, University of Pavia, Pavia, Italy

**Keywords:** micrografts, autologous, rigenera, biomaterial, sinus lift, bone augmentation

## Abstract

Sinus lift augmentation is a procedure required for the placement of a dental implant, whose success can be limited by the quantity or quality of available bone. To this purpose, the first aim of the current study was to evaluate the ability of autologous periosteum-derived micrografts and Poly(lactic-co-glycolic acid) (PLGA) supplemented with hydroxyl apatite (HA) to induce bone augmentation in the sinus lift procedure. Secondly, we compared the micrograft's behavior with respect to biomaterial alone, including Bio-Oss® and PLGA/HA, commercially named Alos. Sinus lift procedure was performed on 24 patients who required dental implants and who, according to the study design and procedure performed, were divided into three groups: group A (Alos + periosteum-derived micrografts); group B (Alos alone); and group C (Bio-Oss® alone). Briefly, in group A, a small piece of periosteum was collected from each patient and mechanically disaggregated by Rigenera® protocol using the Rigeneracons medical device. This protocol allowed for the obtainment of autologous micrografts, which in turn were used to soak the Alos scaffold. At 6 months after the sinus lift procedure and before the installation of dental implants, histological and radiographic evaluations in all three groups were performed. In group A, where sinus lift augmentation was performed using periosteum-derived micrografts and Alos, the bone regeneration was much faster than in the control groups where it was performed with Alos or Bio-Oss® alone (groups B and C, respectively). In addition, the radiographic evaluation in the patients of group A showed a radio-opacity after 4 months, while after 6 months, the prosthetic rehabilitation was improved and was maintained after 2 years post-surgery. In summary, we report on the efficacy of periosteum-derived micrografts and Alos to augment sinus lift in patients requiring dental implants. This efficacy is supported by an increased percentage of vital mineralized tisssue in the group treated with both periosteum-derived micrografts and Alos, with respect to the control group of Alos or Bio-Oss® alone, as confirmed by histological analysis and radiographic evaluations at 6 months from treatment.

## Introduction

The restoration of a partial or complete maxilla without teeth can be achieved via the positioning of dental implants, but this approach is commonly limited in success by the quantity or quality of available bone. Furthermore, to create a space between the maxillary sinus floor and the Schneider membrane, it is necessary to perform a sinus lift augmentation, a procedure first proposed by Boyne and James (1980) and later successfully used for managing cases of posterior maxilla with deficient crestal bone (Del Fabbro et al., 2008).

To date, there are only two main techniques to augment sinus lift: the transalveolar (crestal) and the lateral windows, but several modifications of these techniques have been proposed (Wallace and Froum, 2003; Woo and Le, 2004; Sotirakis and Gonshor, 2005). In this procedure, bone substitutes represent key players to increase the rate of success because they possess biocompatibility, permit bone migration and surface colonization by osteogenic cells, present greater capacity to mimic the physical properties of bone, and are available in a great amount at a reasonably low price (Barradas et al., 2011).

Actually, the bone substitutes used for sinus augmentation are provided as different types of autograft, allograft, alloplastic, and xenograft biomaterials or growth factors and, although histomorphometric studies have reported that the amount of new bone and soft tissue components are not the same when using different biomaterials; the choice of ideal graft material is still controversial. In this study, we propose the use of autologous periosteum-derived micrografts as a new procedure during maxillary sinus lift to improve the rate of success for the placement of dental implants. Autologous micrografts were obtained by the Rigenera® protocol, a new clinical approach that in a short time allows for the obtainment of viable micrografts ready for deployment and that are usable alone or in combination with different biomaterials. To this regard, we previously reported in other studies on the capacity of these micrografts to improve the wound healing of chronic wounds, such as dehiscences (Baglioni et al., 2016; Marcarelli et al., 2016), postoperative wounds (Giaccone et al., 2014), leg chronic ulcers (Trovato et al., 2016; De Francesco et al., 2017), and hypertrophic scars (Svolacchia et al., 2016), when applied alone or in combination with collagen sponges. Furthermore, the ability of these micrografts, in combination with platelet rich plasma (PRP), to induce cartilage regeneration in patients affected by external nasal valve collapse (Gentile et al., 2016) has been reported. Micrograft technology found application in oral-maxillofacial surgery, where micrografts derived from human dental pulp or periosteum were used for periodontal regeneration, bone regeneration of atrophic maxilla, and alveolar socket preservation (Brunelli et al., 2013; Graziano et al., 2013; D'Aquino et al., 2016).

Based on this prior research, the aim of this study was to evaluate the ability of autologous periosteum-derived micrografts in combination with a biomaterial to induce a bone augmentation in the sinus lift during clinical investigation, radiographs, and histologic analysis. In this study, we used a new variant of poly(lactic-co-glycolic) acid (PLGA), comprised of PLGA and porous hydroxyapatite (HA) 20%, commercially named Alos (Allmed, Lissone, Italy), as a biomaterial. Secondly, we wanted to compare the micrografts' behavior with respect to biomaterial alone, including Bio-Oss® (Geistlich Biomaterials, Wolhusen, Switzerland) and Alos.

## Patients and methods

### Ethical considerations

This study is consistent with the ethical principles enunciated by the Declaration of Helsinki and was approved by the Ethical Committee of University of Pavia (minutes March 2014). All the samples evaluated in the present retrospective study were obtained from clinical practice during the implant placement, using a 2.5-mm trephine bur to harvest bone cores in the site chosen for implant installation.

### Patients

A total of 24 patients (12 females and 12 males), ranging in age from 45 to 64 years participated in this study, after signing the informed consent. The inclusion criteria were the following: needing an implant-supported prosthesis; no systemic disease (ASA 1 and 2); no pregnancy (for females); and no routine drug use. All patients were physically healthy, with no underlying systemic disease as determined by medical history screening and no drug interference with osseointegration. Patients were subjected to a professional oral hygiene regimen 1 week before surgery and then performed domiciliary hygiene, consisting of washing the mouth with 0.2% chlorhexidine (CHX) after tooth brushing, twice a day until the time of intervention. The patients were divided into three groups, according to the sinus lift procedure to be performed: group A [Alos + periosteum-derived micrografts (PM)]; group B (Alos alone); group C (Bio-Oss®; Geistlich Biomaterials, Wolhusen, Switzerland, alone). Each group included eight subjects.

### Autologous micro-grafts collection

**Figure 1:**
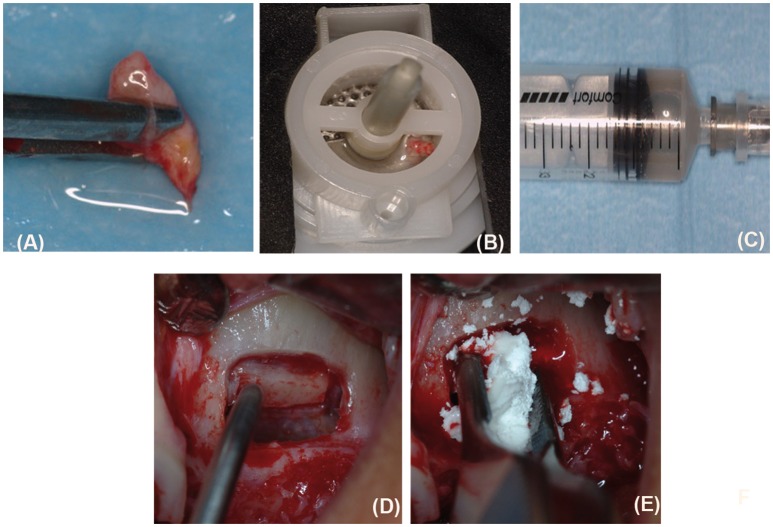
A collection of periosteum-derived micrografts and the grafting procedure. To collect the autologous micrografts, a sample of periosteum **(A)** was inserted in the Rigeneracons device **(B)** and mechanically disaggregated as described in the text. After the disaggregation, the cell suspension containing the micrografts was collected by a syringe **(C)**. On the opened flap **(D)**, we placed PLGA/HA soaked with periosteum-derived micrografts or PLGA/HA or Bio-Oss® alone, as described for each group of study **(E)**.

Periosteum samples harvested on the inner layer of the flap elevated to allow for access to the surgical site were disaggregated by a new medical device called Rigeneracons (Human Brain Wave LLC, Turin, Italy), which is able to filter and select progenitor cells measuring 50 μm in size to create autologous micrografts that can be used without extensive manipulation, in a safe and easy manner, as previously reported (Trovato et al., 2015; Purpura et al., 2016; Monti et al., 2017). Briefly, a 2 mm periosteum sample is harvested from the flap (Figure 1A) and disaggregated using Rigeneracons, with the addition of 1 ml of saline solution (0.9% NaCl; Figure 1B). The mechanical disaggregation is then activated, inserting the device in the Rigenera machine (70 r/min and 15 Ncm) (Human Brain Wave LLC, Turin, Italy) and after 2 min, the micrografts suspension is collected with a syringe using the dedicated hole (Figure 1C). The micrografts suspension is used to soak the biomaterial Alos for 10 min.

### Surgery and grafting procedure

Under antibiotic prophylaxis (2 g amoxicillin and clavulanic acid 2 h before surgery) and disinfection of the oral mucosa by clorexidine digluconate 0.2 %, following local anesthesia (articain 4% with 1:200,000 epinephrine), the flap was elevated (Figure 1D), a lateral bone window was created by piezosurgery (Mectron, Carasco GE, Italy) by means of inserting OT5, and the sinus floor was elevated. Periosteum-derived micrografts were combined with the Alos scaffold in group A, while Alos or Bio-Oss® (Geistlich Biomaterials, Wolhusen, Switzerland) alone were used as the control in the groups B and C, respectively. In group A, the sinus access was closed by a Bio-Gide® Geistlich Biomaterials, Wolhusen, Switzerland membrane seeded with the micrograft suspension (Figure 1E). In groups B and C, the Bio-Gide® membrane (Geistlich Biomaterials, Wolhusen, Switzerland) was used as supplied by the manufacturer. After that, the flap was repositioned using single sutures. Subsequently, after surgery, the patients continued antibiotic therapy for 7 days (specifically, amoxicillin and clavulanic acid 1 g every 12 h for 7 days after surgery) and rinsed with chlorhexidine digluconate 0.2%, twice daily for 4 weeks. The sutures were removed 2 weeks later.

### Radiographic and histological evaluation

After 4 months following the micrograft grafting, the patients were subjected to implant installation with bone biopsy harvesting. A total of five biopsies from treated sites for each group were analyzed and retrieved from dental-implant sites using a 3 mm diameter trephine bur. All samples were fixed in 10% formalin, decalcified in an ethylenediaminetetraacetic acid solution (Osteodec; Bio-Optica, Milan, Italy), and paraffin-embedded. Subsequently, 5 μm–thick-sections were prepared by a microtome (Leica Biosystem, Milan, Italy) and mounted on coated glass slides. Following this, sections were deparaffinized in xylene, immersed in decreasing concentrations of ethanol, and rehydrated in water. Finally, sections were stained with Mallory's trichrome to histologically evaluate the new bone formation. Images were processed with Image J software (http://rsb.info.nih.gov/ij/; National Institutes of Health, Bethesda, MD, USA) in order to measure and compare the size of non-mineralized tissue (NMT) with respect to vital mineralized tissue (VMT) and non-vital mineralized tissue (NMVT) for each histological acquisition.

### Statistical analysis

The statistical significance, where appropriate, was established for *P* ≤ 0.05 using GraphPad 7.0 software.

## Results

**Figure 2:**
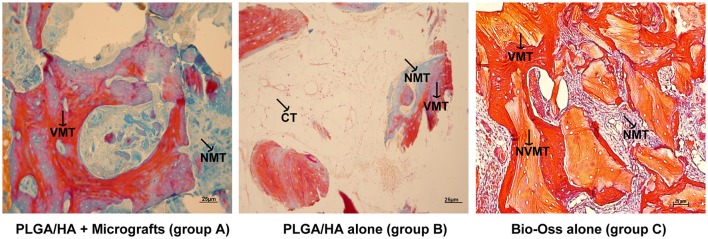
Histological evaluation after 4 months following micrograft application. The biopsy sections for each group of study were stained with Mallory's tricome technique as reported in the text. NVMT, nonvital mineralized tissue (orange); VMT, vital mineralized tissue (red); NMT non-mineralized tissue (blue), CT connective tissue (magnification 40X; scale bar 25 μm).

**Figure 3:**
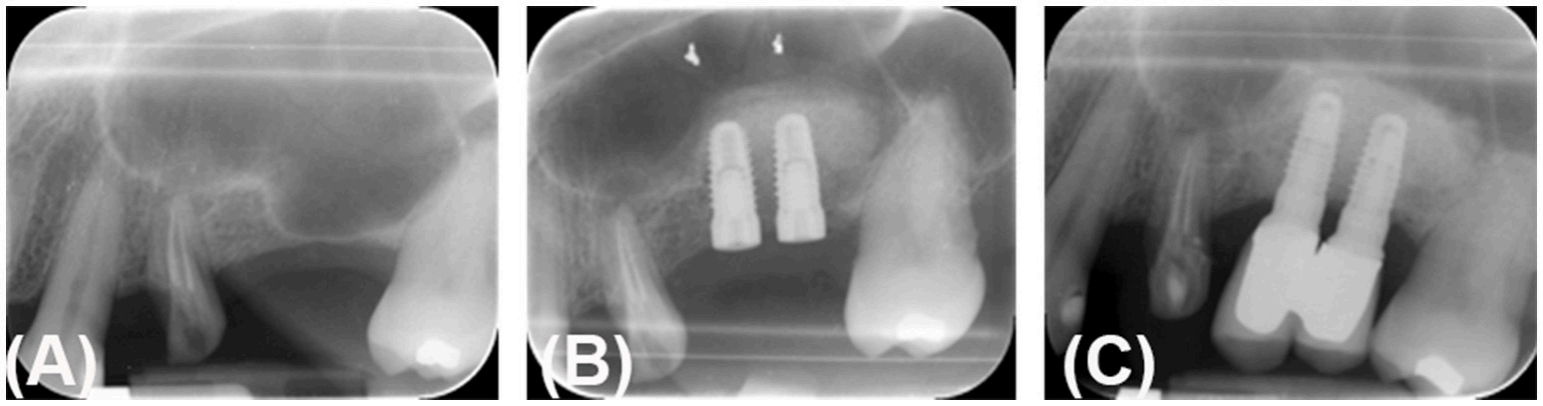
Radiography control after 6 months of micrograft application in patients treated with PLGA/HA + periosteum-derived micrografts. **(A)** Pre-surgical Rx. **(B)** Implant surgery Rx after 4 months and Rx after 2 years **(C)**.

Figure 2 reports the histological findings of bone samples at 4 months after micrograft grafting and comparisons between the new bone formation with respect to either Alos or Bio-Oss® (Geistlich Biomaterials, Wolhusen, Switzerland) alone. It can be observed that in the group treated with periosteum-derived micrografts and Alos (group A), the ossification process was more increased compared to that in the control group B (Alos alone) or in group C (Bio-Oss®; Geistlich Biomaterials, Wolhusen, Switzerland). In fact, in group A, we observed a significant amount of vital mineralized tissue (red) associated with the presence of tissue in the phase of mineralization (blue) and the absence of nonviable mineralized tissue (light orange). On the contrary, in group B, we observed a minor quantity of vital mineralized tissue and more adipocytes, and in group C, we saw a major positivity for nonviable mineralized tissue (light orange) originating from Bio-Oss® action. These evidences were confirmed by histomorphometric analysis reported in Table 1. In fact, we showed that the size of VMT of group A (Alos + micrografts) is higher and statistically significant with respect to mineralized tissue of group B (Alos alone) and group C (Bio-Oss® alone) (*p* < 0.004). Moreover, mineralization in a case of group A can be evaluated by comparing the intra-oral radiographic images taken before the sinus lifting, at the moment of the implant installation, and at 2 years after prosthetical load applied, respectively (Figures 3A–C). The control Rx showed a radio-opacity already at 4 months (Figure 3B) after the implant surgery was performed. After two more months, the prosthetic rehabilitation was finalized and the Rx at 2 years shows an optimal maintenance of the bone level (Figure 3C).

**Table 1:** Histomorphometric analysis.

**Groups**				
**Tissues**	**Alos + micrografts (A)**	**Alos (B)**	**Bio-Oss® (C)**	***p*-value**
VMT	58.5 ± 2.5	20.2 ± 3.1	48 ± 2.5	0.004
NMT	41.4 ± 5.6	5.5 ± 1.6	20.5 ± 3.1	0.003
NVMT	N/A	N/A	31.5 ± 2.3	N/A

*VMT, Vital mineralized tissue; NMT, non-mineralized tissue; NVMT, nonvital mineralized tissue. All values are expressed in % ± SD and calculated by image J software. The statistical significance between three groups was established for P ≤ 0.05 and calculated by Mann-Whitney U-test using GraphPad 7.0 software*.

## Discussion

In this study, we showed for the first time the efficacy of a biocomplex composed by periosteum-derived micrografts and PLGA/HA 20% to augment sinus lift in patients who required dental implants. Furthermore, it was observed that the new bone formation induced by this biocomplex was greater than that by Bio-Oss® (Geistlich Biomaterials, Wolhusen, Switzerland) or PLGA/HA 20% alone.

The role of PLGA/HA in sinus lift grafting is still under debate, and in general there are not sufficient published studies on the effectiveness of this biomaterial in human osseous regeneration. On the contrary, the role of PLGA is well-defined as a delivery system for drugs and therapeutic biomolecules and as a component of tissue engineering applications, because of its biocompatibility and capacity to modify surface properties improving the interaction with biological materials (Gentile et al., 2014).

Bone tissue engineering represents a promising approach in the treatment of pathological conditions in the oral cavity and, to be successful, requires an appropriate source of mesenchymal stem cells, (MSCs), such as dental pulp stem cells (DPSCs), or periosteal stem cells (PSCs), and a suitable scaffold to support their regenerative potential. To this regard, it has been reported that periosteum-derived micrografts express typical markers of MSCs, such as CD73, CD90, and CD105 (Trovato et al., 2015). The positivity of micrografts for MSCs markers was also confirmed for those derived by other tissues, including derma, auricular cartilage, dental pulp, and cardiac atrial appendage (Trovato et al., 2015; Ceccarelli et al., 2017; De Francesco et al., 2017; Monti et al., 2017). Furthermore, to confirm the regenerative action of periosteum-derived micrografts, the ability of periosteal cells to self-commitment toward osteogenic lineage (Colnot et al., 2012; Ferretti and Mattioli-Belmonte, 2014; Lin et al., 2014) and to exhibit MSCs-like properties (Mattioli-Belmonte et al., 2015) has widely been reported.

In this study, we used autologous periosteum-derived micrografts plus PLGA/HA 20%, reporting their efficacy to augment the sinus lift procedure by inducing new bone formation after 4 months from the procedure as indicated by histological and radiographic evaluations. This result is according to previous studies, where different authors have reported reduced bone resorption and enhanced osseous tissue deposition (D'Aquino et al., 2016), and on the efficacy of bone marrow aspirate concentrate and Bio-Oss® (Geistlich Biomaterials, Wolhusen, Switzerland) to increase the bone formation vs. Bio-Oss alone® (Geistlich Biomaterials, Wolhusen, Switzerland) (de Oliveira et al., 2016).

To date, different types of biomaterials have been used for sinus augmentation, and the selection of the ideal graft materials is still debated because of their well-known limitations (Al-Nawas and Schiegnitz, 2014; Kamm et al., 2015; Schliephake, 2015). Several researches have demonstrated that MSCs can be used in maxillary sinus augmentation because of their potential to induce bone regeneration (Razzouk and Schoor, 2012). However, further clinical trials are needed to clearly demonstrate the advantages of a cell-based approach over traditional treatments (Mangano et al., 2015).

Finally, we reported in this study an improvement of the ossification process in the presence of biocomplex PLGA/HA 20% and micrografts with respect to biomaterials alone in comparison with the effect with PLGA/HA 20% or the gold standard Bio-Oss® alone (Geistlich Biomaterials, Wolhusen, Switzerland). Bio-Oss® (Geistlich Biomaterials, Wolhusen, Switzerland) is widely used in sinus lift procedures (Cannizzaro et al., 2009; Inchingolo et al., 2010), and several studies have reported promising results in sinus floor elevation procedures by promoting osteogenesis and showing a very low resorbability (Wallace and Froum, 2003; Traini et al., 2007). PLGA, HA, or their combination have been used extensively as artificial scaffold materials for bone tissue repair; moreover, HA exhibits good biocompatibility and osteoconductivity, and PLGA possess low immunogenicity, good biocompatibility, and suitable mechanical properties (Zhao et al., 2017).

Regarding the efficacy of BioOss® (Geistlich Biomaterials, Wolhusen, Switzerland) and PLGA/HA, in a previous study, we reported on a comparison of the efficacy of PLGA/HA alone in maxillary sinus-lift surgery vs. with deproteinized bovine bone (DBB) in a study that found an equivalent vertical dimension of the regenerated bone, but a reduced density of the bone regenerated using PLGA/HA when compared with the density when using DBB (Rodriguez y Baena et al., 2013, 2017). Another recent study reported that PLGA/HA appeared to be completely replaced by newly formed bone, whereas DBB presented significant amounts of residual graft material (Portelli et al., 2017).

To conclude, we can state that autologous periosteum-derived micrografts improve the bone augmentation in combination with Alos as suggested by our radiographic and histological evaluations. To confirm this, in the literature it has been demonstrated that the incorporation of HA on PLGA nanofibers increased the expression of osteogenic genes as well as the calcium mineralization of human mesenchymal stem cells (Kang et al., 2010; Lee et al., 2010). Furthermore, a comparative study reported that mesenchymal stem cells loaded with HA/TCP (tricalcium phosphate) is a more effective alternative than Bio-Oss® or HA/TCP in inducing bone regeneration (Vahabi et al., 2012).

Within the limitations of the present study, these results are suggest a major use of PLGA/HA composite in combination with autologous micrografts in the sinus lift augmentation, even though more clinical trials are needed to confirm its suitability for this procedure.

## Author contributions

Study conception and design: RR, RD, AG, and AP. Acquisition of data: GCe, AA. Analysis and interpretation of data: SL, GCu. Drafting of manuscript: LT, RR, and AP. All authors contributed to the critical revision of manuscript and approved the final version to be published.

### Conflict of interest statement

The authors RD, AG, and LT are members of Human Brain Wave, the company that developed the Rigeneracons medical device used in the study. The other authors declare that the research was conducted in the absence of any commercial or financial relationships that could be construed as a potential conflict of interest.
